# Alcohol email assessment and feedback study dismantling effectiveness for university students (AMADEUS-1): study protocol for a randomized controlled trial

**DOI:** 10.1186/1745-6215-13-49

**Published:** 2012-07-06

**Authors:** Jim McCambridge, Preben Bendtsen, Marcus Bendtsen, Per Nilsen

**Affiliations:** 1 Faculty of Public Health & Policy, London School of Hygiene and Tropical Medicine, 15-17 Tavistock Place, London, WC1H 9SH, UK; 2 Department of Medicine and Health, Linköping University, 581 83, Linköping, Sweden; 3 Department of Computer and Information Science, Linköping University, 581 83, Linköping, Sweden

## Abstract

**Background:**

Alcohol causes huge problems for population health and for society, which require interventions with individuals as well as populations to prevent and reduce harms. Brief interventions can be effective and increasingly take advantage of the internet to reach high-risk groups such as students. The research literature on the effectiveness of online interventions is developing rapidly and is confronted by methodological challenges common to other areas of e-health including attrition and assessment reactivity and in the design of control conditions.

**Methods/design:**

The study aim is to evaluate the effectiveness of a brief online intervention, employing a randomized controlled trial (RCT) design that takes account of baseline assessment reactivity, and other possible effects of the research process. Outcomes will be evaluated after 3 months both among student populations as a whole including for a randomized no contact control group and among those who are risky drinkers randomized to brief assessment and feedback (routine practice) or to brief assessment only. A three-arm parallel groups trial will also allow exploration of the magnitude of the feedback and assessment component effects. The trial will be undertaken simultaneously in 2 universities randomizing approximately 15,300 students who will all be blinded to trial participation. All participants will be offered routine practice intervention at the end of the study.

**Discussion:**

This trial informs the development of routine service delivery in Swedish universities and more broadly contributes a new approach to the study of the effectiveness of online interventions in student populations, with relevance to behaviors other than alcohol consumption. The use of blinding and deception in this study raise ethical issues that warrant further attention.

**Trial registration:**

ISRCTN28328154

## Background

Alcohol causes huge problems, both for population health and for society more broadly. It is responsible for approximately 4% of the global burden of disease, similar to tobacco, with a greater impact in high-income countries and among men, for example accounting for 11% of all male deaths in WHO European Region in 2004 [1]. Population-level interventions that seek to influence the price, availability and cultural acceptability of hazardous and harmful drinking may be complemented by individual-level brief interventions delivered in health systems and elsewhere [2]. Brief interventions are typically offered opportunistically by non-specialists in routine contacts with patients attending healthcare services for other reasons and take only a few minutes to deliver [2,3].

Evidence for the effectiveness of brief interventions is based on randomized controlled trials and systematic reviews, which have consistently identified small effects on drinking behavior and related problems [4-6]. Although large-scale implementation programs are relatively recent, there have been longstanding difficulties in persuading generic health and welfare practitioners to embrace this work in routine practice [7]. The widespread use of computers and the internet offer other ways to reach large numbers of hazardous and harmful drinkers which overcome implementation problems due to practitioner reluctance to discuss drinking [7]. It may also be cheaper to implement online interventions and more acceptable to those targeted, though these features will not be important to public health unless interventions can also be demonstrated to be effective [8,9].

The research literature in this area is at an early stage of development but is evolving quickly. A number of recent systematic reviews provide preliminary evidence of effectiveness for a range of computerized interventions [10,11]. However, there are also examples of apparently well designed interventions (for example, [12]) not being found to be effective [13]. This highly naturalistic study was undertaken in a web browser population, and interpretation is complicated by the many unresolved methodological problems impeding progress in the evaluation of the effectiveness of online interventions [14]. Careful selection of study populations has been successfully used to demonstrate effectiveness in general population samples [15,16].

University student populations are very prominent in the existing literature, having been relatively extensively studied compared to non-students [11]. Heavy drinking among university students is a seemingly unremarkable and age-old phenomenon that is now globalized [17]. Given the well established role of heavy drinking in student cultures and the extent of internet use among students it is perhaps unsurprising that trials of internet interventions to promote safer drinking have been undertaken [18-20]. There are now effectiveness reviews of normative feedback interventions delivered in various ways among students [21] and of computerized interventions for this population [22].

Most previous student studies have required participants to attend laboratory or other controlled settings, rather than allowing access to interventions using their own computers [10,11,23]. Few of the published studies to date have described projects that have made comprehensive use of electronic media, by recruiting large numbers of participants via email, or allowed participants to engage with interventions naturalistically, when, where and how preferred by the participants themselves. Thus, most of the existing research has been undertaken in relatively artificial efficacy conditions, not closely resembling how interventions found to be effective would be routinely delivered [23].

The key exception to this is the research program on electronic screening and brief intervention (e-SBI) by Kypri and colleagues in Australia and New Zealand. Following earlier studies which recruited participants in student healthcare services [24-26], THRIVE was a large-scale effectiveness trial which invited 13,000 students to participate and subsequently randomized 2,435 risky drinkers to intervention or control conditions, with 2,050 providing follow-up data after either 1 or 6 months or both [27]. After 1 month there was a 17% reduction in alcohol consumption in the assessment and feedback group compared to a non-intervention assessment-only group. This had attenuated to an 11% difference after 6 months [27]. Currently under way in New Zealand are the first multisite large-scale effectiveness trials [8]. There remain many important knowledge gaps concerning the effectiveness of online interventions, including basic questions such as whether effectiveness is established and what effect sizes may be expected, under what conditions, for which populations and with which specific content (for example is feedback required, and if so should it be normative), as well as for different delivery models and across cultures.

Like their peers in other countries Swedish students also drink heavily. One recent study at Linköping University found that heavy episodic (or binge) drinking was normative, being reported by majorities of both sexes and almost three-quarters of all males [28]. The e-SBI model originally developed by the Lifestyle Intervention Research group at Linköping University was originally conceived within an effectiveness framework [29]. It is based upon an initial email to students from the student healthcare service, providing a link to a website for assessment and feedback. The core e-SBI content involves feedback on recommended limits of alcohol consumption and normative comparisons of drinking with Swedish students of the same age and sex. Previous research by this group has shown that this e-SBI model is a feasible way of reaching large numbers of students in ways permitting the conduct of effectiveness trials to evaluate detailed intervention content [29]. The first trial of this intervention found no differences between brief and more extensive normative feedback content though attrition problematically reduced available sample size [30]. Building upon the initial trial we undertook a further study as an unusually large pilot study with outcome data provided by 2,400 students to prepare for the trial described here. A key aim was to improve study retention and the implications of this pilot work for preparation and design of the present study are described in detail in the Methods section (a report is also being prepared for publication).

Among the issues that have presented difficulties in developing this area of research are uncertainties about the most appropriate control groups, due largely to overlap and similarities between feedback and assessment content [31]. Alcohol researchers have long been interested in the possibility that assessment or screening of alcohol consumption per se can reduce drinking [4,32,33]. It has not been unusual to observe reductions in drinking in non-intervention control groups of the order of 20% at later follow-ups [34,35]. These uncertainties have been made explicit by randomized controlled trials demonstrating apparent effects of assessment procedures, including when undertaken online [36] and when limited to screening alone [37]. In the latter case, pen and paper completion of the Alcohol Use Disorders Identification Test (AUDIT), a ten-item alcohol screening questionnaire [38], alone led to self-reported reduced drinking. While this could have been due to the specific effects of answering questions, it could also have been a Hawthorne effect in response to having one’s drinking studied [39], which this group may have inferred while the control group could not have done [37]. This entire literature is necessarily based upon self-reported drinking outcomes. Self-reports have been found to be reliable in alcohol treatment contexts [40] but have been little studied in brief intervention study contexts [41]. Assessment effects have also been conceptualized and studied in different ways in a range of disciplines and fields of research, and these have identified effects upon objectively ascertained outcomes in randomized trials [42-44].

In a recent systematic review of existing trials of randomized evaluations of assessment reactivity in brief intervention studies, effects were found to be of a somewhat smaller magnitude than typical brief intervention effects, hovering around the threshold for statistical significance, and being within the lower end of the confidence intervals for meta analytic estimates of effects [45]. When attention was restricted to university student populations, however, stronger effects were apparent, similar in magnitude to those of brief interventions themselves [45]. If simply by answering questions on one’s drinking, however, does subsequently lead to reduced drinking, large-scale implementation of simple screening surveys as interventions among university students might have a considerable public health potential [29]. This suggests also a need to demonstrate that more elaborate online interventions provide additional effects that are also acceptable to those targeted as well more cost effective than less elaborate interventions. There are as yet sparse data available on costs and cost effectiveness [46].

Making unbiased comparisons to guide decision making about alternative courses of action is the fundamental business of trials and assessment effects may introduce bias in studies of behavior change in ways that are not widely appreciated [47]. Better understanding the effects of assessment per se on drinking behavior should inform thinking both about intervention and control content. Other aspects of taking part in trials, apart from assessment reactivity, may also influence both research participation dynamics and the behaviors being studied and this possibility warrants dedicated studies [48]. For example, precisely what we expect participants to do in trials may well have implications for retention [49].

The overall aim here is to evaluate the effectiveness of e-SBI, employing an RCT design that takes account of baseline assessment reactivity and other possible effects of the research process [48]. Alcohol trials without any form of baseline assessment are rare and this situation hampers evaluation of the true effects of brief alcohol interventions whose content includes assessment [33,45]. Even more rare are studies that eschew typical trial recruitment processes due to concerns about interference with study aims, though they do exist (for example, [50]) and such Zelen designs [51] have been widely used in other areas [52]. The present study design will allow exploration of the magnitude of the feedback and assessment component effects, and is specifically designed to constrain possible effects of research participation on the control group, in testing e-SBI effectiveness.

## Methods/design

### Design

This is a three-arm parallel groups trial in which routine provision of e-SBI (group 1) is compared with assessment-only (group 2) and no contact control (group 3) study conditions. Groups 1 and 2 will complete identical assessments, the sole difference between them being that group 1 will receive normative feedback as usual whereas group 2 will not. Group 3 will only be contacted after 3 months, at which time both groups 1 and 2 also complete outcome data collection.

### Hypotheses

There are four main hypotheses, as follows:1) drinking in groups 1 and 3 will differ, with group 1 drinking less, providing a test of the effects of universal e-SBI provision in an unselected population of university students; (2) drinking in groups 2 and 3 will differ, with group 2 drinking less, providing a test of the effects of assessment-only in an unselected population of university students; (3) drinking in groups 1 and 2 will differ, with group 1 drinking less, providing a test of the effects of adding feedback to assessment-only among those who were risky drinkers participating at study entry; (4) drinking in groups 1 and 2 will differ, with group 1 drinking less, providing a test of the effects of adding feedback to assessment-only in an unselected population of university students.

Three hypotheses concern possible effects in unselected populations, that is, without reference either to drinking behavior or to earlier study participation. It is further hypothesized that these effects will be present among those whose drinking is determined to be potentially hazardous, that is, excluding non-drinkers and very infrequent drinkers (see Outcomes evaluation).

### Participants and setting

The study will be performed simultaneously at two different universities in Sweden, one in the northern part of Sweden, Luleå, and one in the southern part, Linköping. These institutions have been selected on the basis of previously conducted research involving the local student healthcare services who are responsible for alcohol interventions [29,30]. All students at both universities during the autumn 2011 term (that is, in terms 1, 3 and 5) will be included and all will be offered routine e-SBI provision during this term at the end of the study in addition to brief lifestyle feedback provided by the study (see below). Students in a single year group at one of the universities participated in a pilot study a year earlier (see below).

### Randomization and other study procedures

Email addresses will be collected from both official university registers in 3 separate data files, 1 for each year, approximately 15,300 in total. Sequence generation involved each participant being given a random number between 0.0 and 1.0 with 2 decimals in OpenOffice Calc 3.1. All participants have a 1/3 probability of allocation to any particular study condition. Randomization is fully computerized, does not employ any strata or blocks within each year at each university, and is not possible to subvert as all subsequent study processes are fully automated. The initial email to groups 1 and 2 is sent from the student healthcare services as usual, with the restriction that feedback is not offered to group 2. No contact is made at this point in time with group 3.

**Figure 1:**
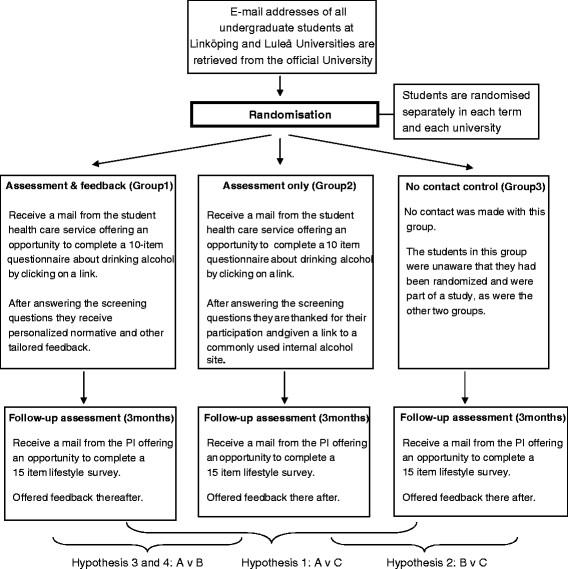
Flowchart for the Amadeus-1 trial.

Then, 3 months later all three groups are sent an identical email by the Swedish Principal Investigator (PB). This makes no reference to alcohol nor to the previous email from the student healthcare services and comprises an invitation to participate in an online lifestyle survey with a 15-item questionnaire. Study drinking outcomes are derived from three questions in this survey. There are then two reminders containing a link to the questionnaire followed by a third reminder with three questions (one on alcohol) embedded in the body of the email. Brief lifestyle feedback is provided to those completing the lifestyle survey. See Figure 1 for an overview of the process.

### Intervention content

Intervention delivery begins with receipt of an email. Every student has a personal university email address that is obligatory to use. All official mails are delivered through this address. The third author (MB) sends out the initial emails to the individual students on behalf of the student healthcare centers. Groups 1 and 2 both complete an alcohol assessment instrument comprising ten items. Group 1 then receives feedback whereas group 2 are simply thanked for their participation and offered a link to a commonly used alcohol website without content understood to be effective in assisting behavior change. The hyperlink contained within the body of the email is no longer valid after completion of the questionnaire when the responses are stored in the study database. This prevents multiple responses, while allowing the questionnaire to be completed in more than one session if required.

Group 1 receives feedback immediately upon completion of the assessment consisting of three statements summarizing their weekly consumption, their frequency of heavy episodic drinking and their highest blood alcohol concentration over the last 4 weeks, comparing drinking patterns against the safe drinking limits established by the Swedish Institute for Public Health [30]. After this follows comprehensive normative feedback with information describing participants’ alcohol use compared to their peers in Swedish universities, and, if applicable, personalized advice concerning the importance of reducing any unhealthy levels or pattern of consumption. The feedback can be printed out by the student. A demonstration version of the assessment and feedback intervention can be viewed at http://demo.livsstilstest.nu. All participants will be offered routine practice alcohol e-SBI at the end of the study within the same term.

### Blinding

Groups 1 and 2 are unaware that they are participating in a research study when they respond to the initial emails. Both groups are given to understand that these emails are provided as routine practice by the student healthcare centers to encourage students to consider their drinking. Thus, all three groups are unaware they are participating in an intervention study and that they have been randomized. Subsequently at follow-up, no explanation of the true nature of the study is given to students. Instead they are invited to participate in a seemingly unrelated cross-sectional lifestyle survey without any particular focus on drinking behavior (see Attrition). As all study procedures are automated, the research team has no direct contact with study participants. The use of blinding and deception in this trial raises ethical issues (see Discussion). The study was approved by the Regional Ethical Committee in Linköping, Sweden (number: 2010/291-31 on 12 October 2010).

### Sample size

The marginal costs involved in increasing the numbers to whom e-SBIs are delivered are negligible. Therefore even very small effects are likely also to be cost effective above the basic threshold cost involved in providing the service. These observations also apply to undertaking research to evaluate effectiveness and suggest that sample size should be as large as possible to detect very small effects. The pilot study indicated that any between-group differences are likely to be very small and it is a moot point as to whether this study should be even larger. Our power calculation assumed no difference between the 2 universities, and a follow-up rate of 50% (see Attrition below; 7,650 of a study population of approximately 15,300). For 3 groups of 2,550 this yields approximately 90% statistical power to detect an effect size of 0.09 standard deviations between any 2 contiguous pairs (group 1 vs group 2 or group 2 vs group 3) or 0.18 between group 1 and group 3. Alternatively we have in excess of 80% power to detect effects of 0.08 and 0.16 standard deviations, respectively.

### Attrition

Attrition has been a major source of difficulty in previous work in developing e-SBI in Linköping and other Swedish universities [30]. It is also a significant problem in the conduct of online trials in other populations [14]. The initial take-up of the routine service provision of e-SBI has varied between 10% to 60% in different universities due to varying patterns of email use and rates of hazardous drinking, as well as the salience of alcohol and interest in intervention. In the selected universities take-up rates have been consistently around 40%. In previous follow-up studies, less than half of those who participated at baseline did so at first follow-up and approximately one-quarter participated in second follow-ups [29,30].

A different approach was taken in the pilot study to address the issue of attrition within the same three-group design structure as outlined here (our forthcoming report will contain further details). Rather than follow-up emails being sent by the student healthcare service as was performed previously, blinding of participants to trial conduct was implemented. This involved an explicit attempt to separate the experience of follow-up from earlier e-SBI delivery. An email was sent by the second author (PB) requesting participation in a survey of student alcohol consumption, partially following the approach of Kypri and colleagues who invited participation in a series of surveys at the outset and who obtained high follow-up rates [27]. Incentives in the form of cinema tickets were also offered in the pilot study [53].

This was only partially successful. Participation rates in ‘follow-up’ were slightly higher than at baseline (approximately 41% compared to 37%) in the two groups randomized to earlier contact. This comprised both the involvement of some who had not previously participated as well as attrition among some of those who had. While this measure had restricted the reduction in follow-up seen previously, it introduced a new problem; differential participation by group 3 (approximately 52%) comprised the equivalence of the three groups. By virtue of randomization, there was a strong basis for inferring that this could only have been caused by the earlier involvement with the study. The earlier invitation to participate in the alcohol e-SBI was not sufficiently different from the later alcohol survey. To rectify this, we decided for the main trial to abbreviate the alcohol outcome measures and conceal them within a lifestyle questionnaire in the follow-up study. It was reasoned that we had simply not gone far enough with our earlier attempt. Note that this extends blinding to a specific focus on alcohol at the point of outcome data collection. We will also add a third reminder at follow-up with the option of completing three items in the body of the email (as well as the hyperlink).

### Outcomes evaluation

Intention-to-treat analyses are primarily used in clinical trials to address problems with lack of compliance with allocated interventions [54,55]. In the present context the intervention comprises an automated email providing a means of accessing a website in an unselected population in which the prevalence of hazardous and harmful drinking is elevated. Lack of take-up of intervention is arguably more fundamentally a matter of reach rather than of effectiveness here, as there are not noteworthy costs associated with lack of take-up, though this situation does complicate evaluation of effectiveness and it is recommended that an attempt is made to account for all randomized participants [55]. The intervention could be defined more narrowly as delivered to those who access the website, with the email merely being the means of recruitment. Even if this definition is applied, the intervention will still be accessed by students whose drinking is not risky and who would thus not be deemed to merit individual targeting for intervention. More narrowly still, outcome evaluation could be restricted to those whose drinking is found to be risky. The overarching problem is that a greater number of people are randomized than would be targeted for intervention. Consideration of outcomes evaluation needs to take account of these issues.

By necessity, inferences involving group 3 can only be drawn in entirely unselected populations, as there are no baseline data with which one might construct subgroups randomized or otherwise for comparative purposes. Hence the form of hypotheses 1 and 2, which should be noted as highly conservative approaches to outcome evaluation as they will unavoidably include data that will bias the findings towards the null (both from non-participants at baseline and those who are not risky drinkers). To address this problem, analyses shall also be undertaken which exclude those determined in follow-up data to be unlikely to have been hazardous drinkers at study entry. Specifically non-drinkers and very infrequent drinkers (reporting never drinking or monthly or less frequently to AUDIT-C item 1; see below) will be excluded. This is also somewhat conservative to the extent that intervention effects may lead participants to drink rarely or not all but this does help with the problem previously described.

Hypotheses 3 and 4 concern only groups 1 and 2 and provide alternative ways of evaluating the specific effects of feedback. Hypothesis 3 is preferred as it addresses this question among the most relevant subpopulation of risky drinkers exposed to intervention who also later participated in follow-up and this will be the primary analysis for this question. It is acknowledged that the departure from intention-to-treat (ITT) implies a risk of bias. It is also judged that methods such as complier-average causal effect analysis are inappropriate due to the unusual study context. This reasoning for preferring a per protocol analysis over an intention-to-treat one has previously been applied in online alcohol trials [9].

### Outcome measures

Three items within the 15-item survey instrument are dedicated to assessment of drinking, alongside questions on smoking, diet and physical activity and sociodemographic characteristics. These items are the three questions of the AUDIT-C [56]. The two primary outcomes are AUDIT-C scores and the proportions of risky drinkers (according to the Swedish definition [57]). The three secondary outcomes are the component items of the AUDIT-C; number of heavy episodic drinking episodes per month, frequency of drinking and typical quantity consumed. The psychometric properties of these data when administered online have been found to be reliable in similar student populations [56,58].

### Data analyses

Intention-to-treat analyses in relation to hypotheses 1, 2 and 4 will be undertaken among all those providing follow-up data without any imputation for missing data. The only additional analyses here will consider how informative are patterns of response to reminders in relation to those not participating at follow-up and no attempt will be made to account for the randomized populations as a whole [55]. The per-protocol analysis for hypothesis 3 will be undertaken among all those in groups 1 and 2 who are risky drinkers at baseline who participate in follow-up. We will also include baseline measures of risk as covariates and examine patterns of attrition in these two groups and their possible impact on findings. We will undertake exploratory analyses of possible effect modification by university, term, faculty, age and gender. Effect sizes will be calculated as standardized mean differences or as ratio measures of effect. Student t tests and *χ*^2^ tests will be used supplemented by regression-based analyses as needed. All analyses will conform to a prespecified plan with data transformations appropriate for skewed data to be determined. There will be no interim analyses or stopping rules.

### Focus group substudy

Guidance on the use of deception in research indicates that debriefing of participants occur as soon as practically possible (for example, [59]). This is usually simple and practical with relatively small numbers in psychology laboratories and other settings in which deception is usually used. While we agree that is important that debriefing should be done, it is not clear, however, how it should be done in the context of an online study such as this with large numbers of participants. For example, if we did this by email and received a small number of extremely unhappy comments, it is not obvious how these should be interpreted or handled. For these reasons we plan to defer debriefing all participants until after we explore in-depth participant views on the acceptability of the deception used and on appropriate debriefing methods. We will convene focus groups for this purpose. At the end of follow-up, we will ask all participants for a phone number for recruitment to a focus group interview on participation in research. Assuming numbers permit, we will randomly select participants to run two focus group sessions, one at each university, aiming for five participants each from groups 1 to 3 in both focus groups.

## Discussion

Universities have an obligation to create an environment where student drinking does as little harm as possible, both for the students’ short-term well being and educational attainment and to mitigate longer-term societal consequences. There are approximately 100,000 new students entering universities in Sweden each year. The Swedish National Institute of Health has decided to implement e-SBI in all universities in Sweden but the effectiveness of such interventions offered in this way is still unclear, as is the extent to which effectiveness may be specifically the product of feedback rather than the assessment components. These Swedish developments have been informed by, and themselves inform, the rapidly evolving research and practice contexts in other countries.

The main methodological innovations presented here concern the randomization and complete non-contact of the control group at baseline and the nature of the blinding practiced at follow-up in order to constrain differential attrition. Randomization of participants in excess of those usually targeted for intervention presents inferential difficulties. In the present study an unusual opportunity to dismantle existing routine practice in interventions delivery and also to omit usual trial entry procedures for individually randomized trials, for both substantive intervention effectiveness evaluation and for methodological purposes. Consideration will later need to be given to the value of this unusual research design as well as to possible alternatives such as cluster randomization.

This study addresses quite a number of significant methodological challenges. We aim to email approximately 15,300 individuals and recruitment and retention of participants equivalently between groups on this scale has not previously been attempted in a brief alcohol intervention trial to our knowledge. Outcomes evaluation is complex and successfully and reliably capturing any small effects that do exist is highly relevant to large-scale public health endeavors to influence health-compromising behaviors.

The methodological basis of decisions to implement blinding and deception has been outlined above, with the former characterizing baseline contacts and the latter involved in follow-up. Similar decisions have been made by Kypri and colleagues for similar reasons, the small effects under study being judged likely to be adversely impacted by research participation artifacts which will introduce bias [8]. This methodological imperative runs counter to the ethical imperative of informed consent. We believe these issues are sufficiently important to warrant extended consideration and are writing a paper that outlines our thinking about the ethical issues involved. We have also incorporated a focus group substudy here for in-depth debriefing on the nature of the deception used, exploration of participant responses, and to aid decision making about the methods and content of debriefing. Methodological progress in this area of work needs to proceed in tandem with the development of ethical considerations.

## Trial status

At the time of submission participants had been randomized and groups 1 and 2 had received the initial emails. No participants had yet received the invitations to participate in the seemingly unrelated cross-sectional lifestyle survey.

## Competing interests

The authors declare that they have no competing interests.

## Authors’ contributions

JM and PB had the original idea for the study, obtained funding and led on its design supported by MB and PN. PB has overall responsibility for study implementation. MB does all computer programming associated both with interventions delivery and study data collection. JM wrote the first draft of the study protocol to which all authors contributed. All authors read and approved the final manuscript.
